# Dissociation between Dorsal and Ventral Posterior Parietal Cortical Responses to Incidental Changes in Natural Scenes

**DOI:** 10.1371/journal.pone.0067988

**Published:** 2013-07-09

**Authors:** Lorelei R. Howard, Dharshan Kumaran, H. Freyja Ólafsdóttir, Hugo J. Spiers

**Affiliations:** 1 Institute of Behavioural Neuroscience, Research Department of Cognitive, Perceptual and Brain Sciences, Division of Psychology and Language Sciences, University College London, London, United Kingdom; 2 Institute of Cognitive Neuroscience, University College London, London, United Kingdom; Cardiff University, United Kingdom

## Abstract

**Background:**

The posterior parietal cortex (PPC) is thought to interact with the medial temporal lobe (MTL) to support spatial cognition and topographical memory. While the response of medial temporal lobe regions to topographical stimuli has been intensively studied, much less research has focused on the role of PPC and its functional connectivity with the medial temporal lobe.

**Methodology/Principle Findings:**

Here we report a dissociation between dorsal and ventral regions of PPC in response to different types of change in natural scenes using an fMRI adaptation paradigm. During scanning subjects performed an incidental target detection task whilst viewing trial unique sequentially presented pairs of natural scenes, each containing a single prominent object. We observed a dissociation between the superior parietal gyrus and the angular gyrus, with the former showing greater sensitivity to spatial change, and the latter showing greater sensitivity to scene novelty. In addition, we observed that the parahippocampal cortex has increased functional connectivity with the angular gyrus, but not superior parietal gyrus, when subjects view change to the scene content.

**Conclusions/Significance:**

Our findings provide support for proposed dissociations between dorsal and ventral regions of PPC and suggest that the dorsal PPC may support the spatial coding of the visual environment even when this information is incidental to the task at hand. Further, through revealing the differential functional interactions of the SPG and AG with the MTL our results help advance our understanding of how the MTL and PPC cooperate to update representations of the world around us.

## Introduction

Our ability to learn, recall and navigate large-scale space is thought to rely on a network including the posterior parietal cortex (PPC), retrosplenial cortex and medial temporal lobe (MTL) [1]–[6]. Among these regions, the PPC has been implicated in egocentric spatial processing (e.g. [1], [7]). However, the contribution of different subregions within PPC to processing topographical stimuli remains unclear. Some neuroimaging studies find increased activity in the angular gyrus (AG) [8]–[10], others find increased activity in superior parietal gyrus (SPG) [4], [11], while several report co-activation of AG and SPG [12]–[20].

Previous neuroimaging work has elucidated the role of the PPC in visual attention [21], [22]. These studies have provided evidence that a dorsal system (including the SPG) provides top-down control of visual attention and a ventral system (including the AG) supports bottom-up stimulus detection and re-orienting to salient events [21]–[24]. Recent work also suggests that such a dorsal/ventral division may also apply to episodic memory processes [25]–[37].

Here we use an fMRI adaptation (fMRA) approach to probe the nature of information represented within regions of the PPC. Whilst fMRA has been widely used to characterize the neural representations and computations in regions within the ventral visual stream (e.g. [38]) and more recently the MTL (e.g. [39]), this technique has been less often used to study the nature of information processing carried out by the PPC (although see e.g. [40]). Whilst an early study [41] which used a broadly related approach (i.e. oddball paradigm) observed both object and location coding in the PPC, it did not illuminate a putative dissociation between the contribution of different posterior parietal regions (e.g. AG vs SPG), nor exclude the possibility that the observations could reflect coding of surprise engendered by the occurrence of oddballs.

In recent work we used fMRA to explore the response of MTL regions to change in natural scenes and a parallel eye-tracking control study to examine saccadic responses to the same stimuli [42]. We reported a double dissociation between the parahippocampal cortex and the hippocampus, with the former responsive to change in the scene content and the latter responsive to a spatial change in the scene content. Here, by applying a set of new analyses to these data, we ask three main questions: firstly, what kind of information is coded within the PPC? Secondly, do different regions within the PPC (i.e. AG and SPG) code information in a similar fashion? Thirdly, does the functional connectivity between individual posterior parietal regions and the MTL differ during novelty processing? Despite recent evidence of dissociable connectivity between parietal regions and the MTL, both anatomically [43] and functionally during resting/default states [44]–[47], there has been little examination of the functional connectivity between these regions during the processing of topographical stimuli. As such, understanding how parietal and MTL regions interact is important for constraining models in which they jointly support novelty processing [48], memory encoding and retrieval [26], [30], [33], and spatial memory [2].

We report a dissociation between the AG and the SPG: while the SPG was purely responsive to spatial change (i.e. and not to scene novelty), we find that the maximal response of the AG was to scene novelty – findings that cannot be easily explained by differences in eye movements obtained in a separate behavioural study. We also observed an increase in functional connectivity between the AG and parahippocampal cortex in relation to scene novelty. Independent of this novelty response, increased activity in both AG and parahippocampal cortex was associated with subsequent familiarity for scenes re-presented post-scan. Our findings provide new insights into the types of neural representations supported by different regions within the PPC, and the nature of their interactions (i.e. functional connectivity) with regions within the MTL.

## Materials and Methods

### Experiment 1: fMRI

The present study provides novel analyses of a previously published dataset. All aspects of the experimental materials and methodology are identical to those described in detail in the previously published manuscript; hence, we refer the reader to Howard et al. [42] for a full description of this section. Here, we provide a brief summary of the key aspects of the experimental materials and methods in addition to a detailed description of the new fMRI data analyses.

#### Participants and ethical approval

Twenty two right-handed, healthy volunteers (11 males) with normal or corrected-to-normal vision gave informed consent to participate in this experiment. Prior to data analyses, two participants (1 male, 1 female) were excluded due to excessive head-movement during scanning. This study was approved by the local research ethics committee at the Birkbeck-UCL Centre for NeuroImaging, London, UK.

#### Stimuli

**Figure 1:**
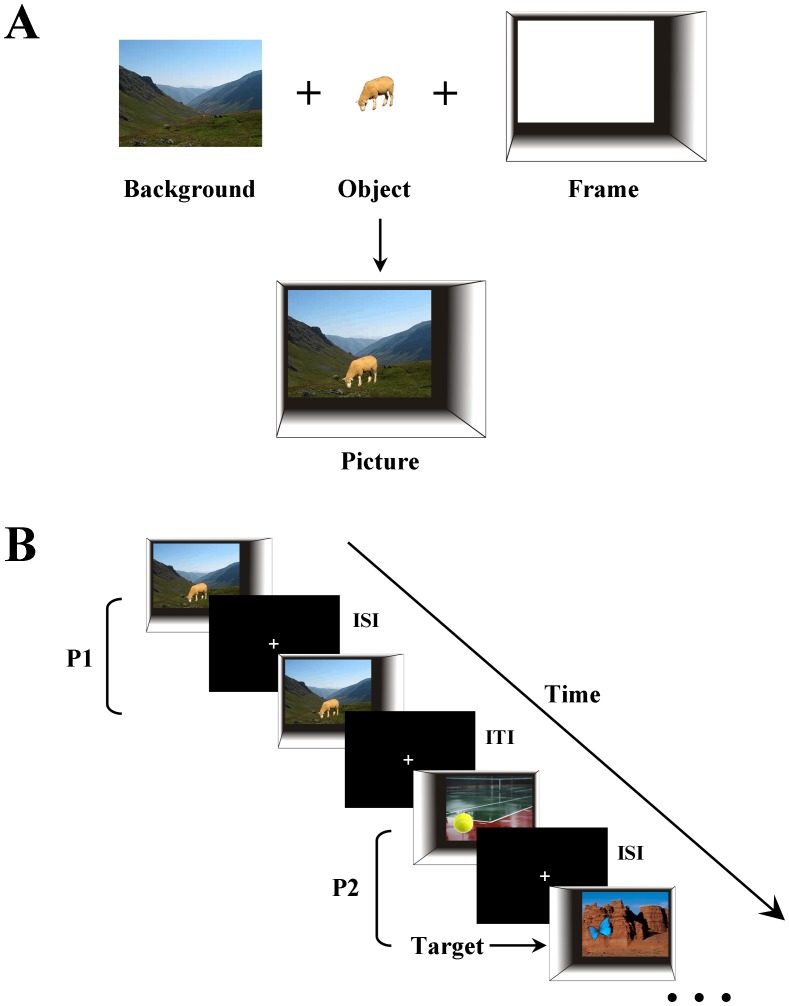
Experimental design. **A**, Coloured picture stimuli comprised the conjunction of objects and background images, embedded within a greyscale frame. **B**, During scanning picture stimuli were presented to subjects in pairs (P1 and P2). Each picture within the pair was presented for 1500 ms, separated by a 250 ms inter-stimulus interval (ISI). An inter-trial interval (ITI) of 2000 ms separated each pair. During all intervals a black screen, with a centrally presented white fixation cross, was shown. Subjects performed an incidental target (butterfly) detection task throughout the experiment; an example of which is shown in P2.

The stimuli used in this study were 289 coloured pictures, containing unique object and background combinations. These combinations (‘scenes’) were inserted into ‘frames’ to create stimulus ‘pictures’ (Fig. 1A). Within scenes, salient objects were paired with backgrounds so that they were contextually congruent (i.e., boats were positioned on water, and planes in the sky). The vertical position of the object was consistent with the scene (e.g. placing a dog on the beach rather than in the sky) and was not manipulated experimentally. The horizontal position of each object was controlled to one of three positions in its background (left, central or right) and was manipulated experimentally (see below).

#### Experimental design and procedures

**Figure 2:**
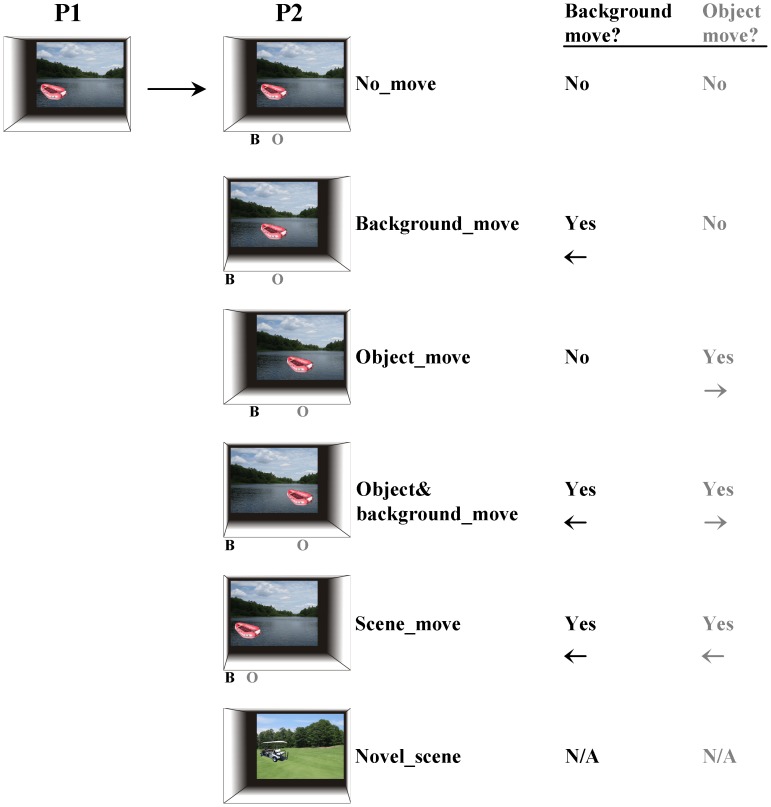
Experimental conditions were created by manipulating the second picture presented. These are illustrated here using one picture, a red inflatable boat on a lake. The position of the object (highlighted by the light grey ‘O’) and the background image (highlighted by the black ‘B’) were manipulated independently to create 5 conditions, these were: 1) there was no movement of any element of the picture (‘No_move’), 2) the background changed to a new position horizontally left or right of where it was previously located on the projection screen (‘Background_move’), 3) the object changed to a new position on the projection screen, moving horizontally left or right of where it was previously located (‘Object_move’), 4) the background and the object both changed to a new position, horizontally left or right of where they were previously located, with the each re-locating in the opposite direction (‘Object&background_move’), or 5) the whole scene (object and background) moves left or right (‘Scene_move’). Also included was a condition in which a completely new object and background was presented as the second picture (‘Novel_scene’). There was one further condition (not shown), the Repeat_scene condition, in which a previously seen scene was re-presented.

Experimental trials comprised pairs of sequentially presented pictures (Fig. 1B). To explore different types of novelty a number of experimental conditions were created by manipulating the second picture presented (Fig. 2). For associative novelty we horizontally manipulated the position of the object and the background independently to create 5 conditions (each 40 trials). For each of these conditions we ensured that changes in the positions of both the objects and the backgrounds occurred equally towards the left and the right. We also ensured that subjects were unable to predict the direction of movement of the object and/or background and were, thus, unable to predict the trial type. For scene novelty we created a condition in which a completely new object-background combination was presented in the second picture (‘Novel_scene’). A condition in which scene novelty was diminished was created by repeatedly presenting a familiar scene, without any associative changes (‘Repeat_scene’). There were 20 trials of the Novel_scene and Repeat_scene conditions. Finally, as part of our incidental target detection task we included target pictures (24 trials) (Fig. 1B). When participants encountered a target picture (containing a butterfly) they were required to press a button with their right index finger. The study was a within-subjects design and experimental trials were presented in a subject-specific, pseudorandom order with the constraint that no more than two trials of the same type were viewed consecutively.

#### Post-scan memory task

Immediately after scanning, we assessed participants’ familiarity with the background images and cued object recall with a surprise memory task. During this task all backgrounds viewed during scanning were re-presented in a fully randomised order. First, participants were asked whether they thought the background image was familiar, then they were asked to recall the object that had been previously presented with the background. Due to the large number of scenes used (289) no lure items were included.

#### Functional MRI parameters and acquisition

We scanned participants using a 1.5 T Siemens (Siemens Medical Systems, Erlangen, Germany) Avanto MRI scanner, with a 32 channel head coil at the Birkbeck-UCL NeuroImaging (BUCNI) Centre. In total, 1288 functional scans were acquired for each participant using a gradient-echo echoplanar imaging (GE-EPI) sequence (TR = 3000 ms, TE = 48 ms, 205×205 FOV, 64×64 matrix). In each volume, 36 oblique axial slices, approximately parallel to the hippocampus and 3.2 mm thick were acquired. A high-resolution T1 structural scan was also acquired for each participant (MPRAGE, 176 slices, 1×1×1 mm resolution).

#### Functional MRI statistical analysis

Statistical parametric mapping (SPM8; http://www.fil.ion.ucl.ac.uk/spm/software/spm8) was used for spatial preprocessing and subsequent analyses. After standard SPM preprocessing, the spatially realigned, smoothed (8 mm FWHM Gaussian kernel filter), normalised (to a standard EPI template in Montreal Neurological Institute (MNI) space) functional imaging data were entered into two voxel-wise subject-specific general linear models.

The first model included 7 regressors of interest: No_move, Object_move, Background_move, Object&background_move, Scene_move, Novel_scene, Repeat_scene. One regressor of no interest, coding target trials, was included. All of these were event-related regressors (stick functions, duration = 0 seconds), the onsets of which were fixed to the presentation time associated with the first picture in each stimulus pair. Each of the regressors was convolved with the canonical haemodynamic response function (HRF). Furthermore, six subject-specific movement parameters (derived from the realignment phase of preprocessing) were also included as regressors of no interest in each model. We used a high pass filter with a cut-off of 128 s to remove low-frequency drifts. Temporal autocorrelation was modelled using an AR(1) process. At the first level, linear weighted contrasts were used to identify effects of interest, providing contrast images for group effects analysed at the second (random-effects) level. The MarsBar SPM toolbox (http://marsbar.sourceforge.net/) was used to extract mean responses from single subjects using defined regions of interest (10 mm spheres) in the AG and SPG [41].

Given our *a priori* anatomical hypotheses, we report activations in the AG and SPG at a threshold of *p<*0.001 (uncorrected for multiple comparisons) and minimum of 10 contiguous voxels. For these regions we also employed a small volume correction (10 mm sphere) located at specific Montreal Neurological Institute (MNI) coordinates on the basis of a prior study [41] examining item and spatial novelty for scenes. These were [−37, −77, 31] and [37, −77, 31] for the left and right AG, respectively, and [24, −59, 57] for the right superior parietal gyrus. These prior coordinates were converted from the original Talairac coordinates using a conversion developed by [49]. We report activations outside these regions at a threshold of *p<*0.001 (uncorrected).

The second analysis of these data was conducted to examine whether we could identify a neural correlate for viewing scenes that were subsequently classified as familiar. Using the data from the post-scan memory task, each picture viewed during scanning was classified according to whether the background scene was subsequently classed familiar or unfamiliar and also whether the object that accompanied this background scene was correctly recalled, or not. Again, the smoothed, normalised functional imaging data were entered into a voxel-wise subject-specific general linear model. In this second analysis there were two regressors of interest: one for pictures where the background scene was subsequently classed ‘Familiar’, and another for pictures where the background scene was subsequently classed ‘Unfamiliar’. There were two regressors of no interest: one for ‘Repeat_scene’ trials, which were used as practice examples in the post-scan memory task and therefore had no contribution to the ‘Familiar’ or ‘Unfamiliar’ regressors, and another that coded target trials. A further subject (female) was removed from this functional imaging analysis as she did not take part in the scene familiarity section of the post-scan memory task. The same general linear model approach was taken with this second analysis.

Given *a priori* anatomical hypotheses, we report activations in the parahippocampal cortex at a threshold of *p<*0.001 (uncorrected for multiple comparisons) and minimum of 10 contiguous voxels. For this region we also employed a small volume correction (10 mm sphere) located at specific Montreal Neurological Institute (MNI) coordinates on the basis of a prior study that examined subsequent memory for scenes (Brewer et al., 1998). These were: [−28, −42, −10] for the left parahippocampal cortex, and [25, −37, −18] for the right parahippocampal cortex [50]. As above, these prior coordinates were converted from the original Talairac coordinates using a conversion developed by [49]. For completeness, we report activations outside these regions at a threshold of *p<*0.001 (uncorrected). It is worth acknowledging that a similar analysis could also have been conducted for the object recall data from the post-scan memory task, however, for many of the subjects there were not enough data points (i.e., successfully recalled objects) to run a robust functional imaging analysis.

Finally, given evidence of direct anatomical connections between the human AG and parahippocampal cortex [43] we reasoned that during our scene processing task we would see increased functional connectivity between the parahippocampal cortex and AG, but not between the parahippocampal cortex and the SPG. We explored the connectivity between these regions in the scene novelty (Novel_scene>Repeat_scene) and spatial change novelty ((Background_move+Object_move+Object&background _move)>No_move) contrasts by computing two psychophysiological interaction analyses (PPI, [51], [52]). Each PPI analysis employed 3 regressors: 1) representing the deconvolved activation time course in a given volume of interest (the physiological variable), 2) representing the contrast of interest (the psychological variable), and 3) representing their cross-product (the psychophysiological interaction term). Both analyses focused on one particular brain region observed in the Novel_scene>Repeat_scene contrast from the first group analysis, i.e., the right parahippocampal cortex [33, −37, −17]. For each participant, we extracted the deconvolved time course of activity in a ROI (a 6 mm radius sphere centred at the voxel displaying maximum peak activity in the group analysis). The time course of activity was corrected for the effect of interest. We then calculated the product of this activation time course with a condition-specific regressor, probing the contrast of interest to create the PPI term. The contrast of interest differed for each of the two PPI analyses run, in the first we used the scene novelty (Novel_scene>Repeat_scene) contrast while in the second we used the spatial change novelty contrast ((Background_move+Object_move+Object&background _move)>No_move). PPI general linear model analyses were then carried out for each subject, and entered into a random effects group analysis.

To further assess connectivity between the parahippocampal cortex and the two posterior parietal regions we ran two additional PPI analyses. The same methods as described above were employed; however, the ROIs used to extract the deconvolved time course of activity and the contrasts of interest used to specify the PPI term differed. Each analysis focused on a particular brain region observed in the Familiar>Unfamiliar contrast from the second group analysis. These were the left [−36, −40, −14] and the right [30, −34, −20] parahippocampal cortex. The contrast of interest was the same for each of the two PPI analyses run (Familiar>Unfamiliar), the same statistical thresholds used in previous analyses were applied to these results.

As with the first set of GLM analyses, we report activations in the AG and SPG at a threshold of *p<*0.001 (uncorrected for multiple comparisons) and minimum of 10 contiguous voxels. For these regions we also employed a small volume correction (10 mm sphere) located at specific Montreal Neurological Institute (MNI) coordinates on the basis of a prior study [41] examining item and spatial novelty for scenes. These were [−37, −77, 31] and [37, −77, 31] for the left and right AG, respectively, and [24, −59, 57] for the right superior parietal gyrus. These prior coordinates were converted from the original Talairac coordinates using a conversion developed by [49]. For completeness, we report activations outside these regions at a threshold of *p<*0.001 uncorrected.

### Experiment 2: Eye-tracking

This experiment provides novel analyses of a previously published dataset [42]. To summarise, Experiment 2 examined the pattern of eye-movements subjects made during our task and explored the possibility that our fMRI results from Experiment 1 might be related to differences in eye-movement patterns. For example, observing a novel scene, or an object in a novel position, might lead to a greater number of saccades executed to explore the scene. In Experiment 2 eye-tracking data were collected during the presentation of the same experimental stimuli and task with a separate, naïve participant group. This study was also approved by the local research ethics committee at the Birkbeck-UCL Centre for NeuroImaging, London, UK. We refer the reader to Howard et al. [42] for a full description of the experimental materials and methodology for Experiment 2. Here, we limit our description to key details regarding data collection and data analysis.

#### Eye-tracking data analysis

**Figure 3:**
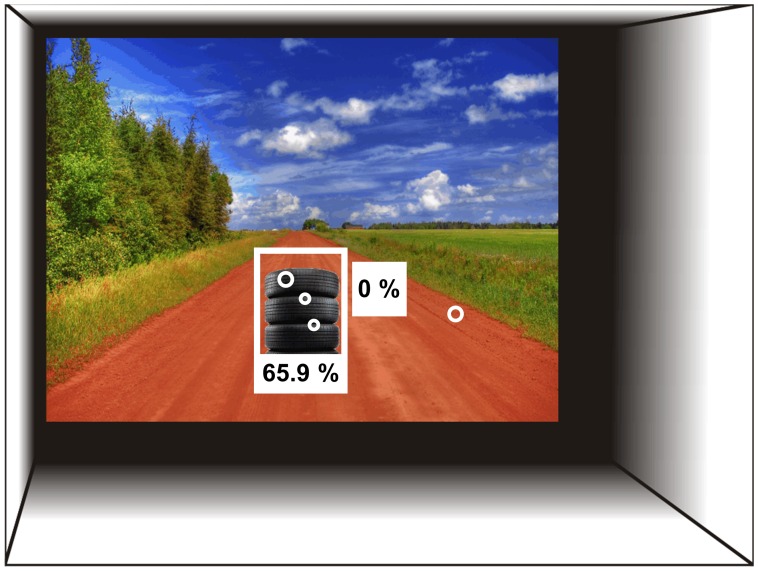
Eye-tracking analysis. The regions of interest (rectangular boxes) used for data analysis were: 1) the current object position, 2) the position of pre-stimulus fixation cross, and 3) the remainder of the scene (background). Fixations (overlaid circles), along with their durations, are shown. For this trial, three fixations were recorded on the current object region and accounted for 65.9% of the total viewing duration, the remaining fixation (34.1% of duration) occurred on the background. No fixations fell within the position of the pre-stimulus fixation cross.

Only data collected during presentation of the second picture were analysed. The inter-picture interval was too short (250 ms) to provide a sufficient duration for a new saccade to be initiated. Thus, to remove noise generated by lingering fixations from the first picture, the initial 350 ms of the presentation of the second picture was removed from the analysis. This time period (350 ms) was selected because it corresponds to the average time needed to execute a saccade (see e.g. [53]). Two analyses were applied. The first analysis examined two measures across all conditions, these were: (1) the mean total number of fixations and (2) mean saccade amplitude (in degrees of visual angle). The second analysis examined whether fixations were located within the region of (1) the object (by using a rectangular box 120% the size of the object) or (2) the background (the remainder of the scene after accounting for the region of the pre-stimulus fixation cross) (Fig. 3).

## Results

Here we present the results obtained from analyses of fMRI (Experiment 1) and eye-tracking (Experiment 2) analyses that are pertinent to the function of the PPC – we refer the reader to a previously published report which details findings from this experiment relating to the hippocampus and parahippocampal cortex [42].

### Experiment 1: fMRI

#### Behavioural results

As reported in Howard et al. [42], participants performed the incidental target detection task during scanning with 96.0% accuracy (SD, 9.2%), mean reaction time of 819 ms (SD, 118.4 ms). Post-scan, participants classified an average of 34.7% (SD 17.7%) of backgrounds as familiar and were able to correctly recall an average of 5.9% (SD 3.54%) of objects. It should be noted, however, that these are uncorrected hit rates - lures were not presented in the recognition memory test due to the large number of studied scenes (see Methods). For 19 subjects we were able to collect scene familiarity judgements and for 20 subjects object recall data. See Howard et al. [42] for a breakdown of performance across conditions. These familiarity and recall scores were significantly positively correlated (r = 0.66, *p = *0.002). We found that the number of backgrounds judged familiar by subjects did not differ statistically across our conditions (F_(5,90)_ = 2.28, *p = *0.053), but the number of objects recalled did (F_(5,95) = _3.68, *p = *0.004). Post-hoc tests revealed that this difference was driven by significantly more objects recalled from the Object_move condition being remembered than the Novel_scene condition (p = 0.046).

#### Neuroimaging results

**Figure 4:**
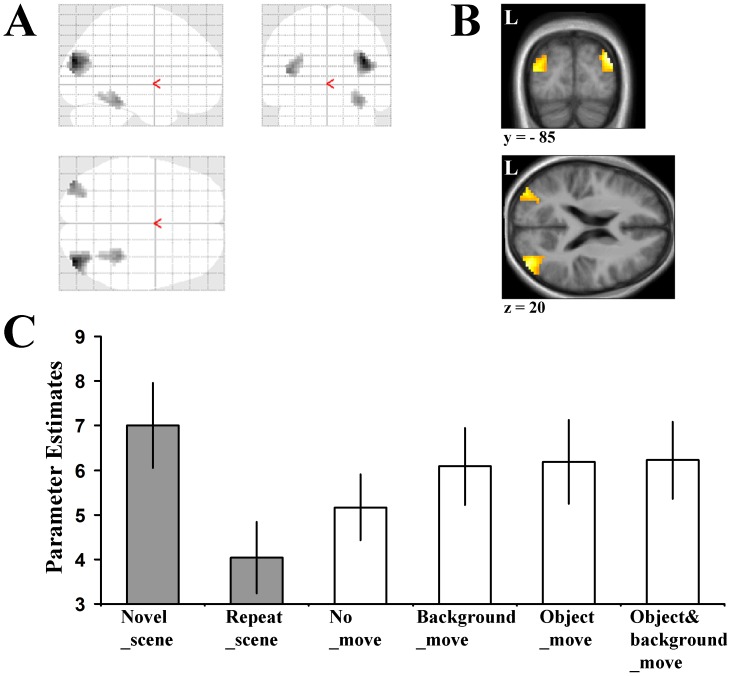
Angular gyrus responds to scene novelty. Increased activity was observed bilaterally in the angular gyrus when the Novel_scene and Repeat_scene conditions were contrasted (Novel_scene>Repeat_scene). The glass brain (**A**) along with Coronal and Axial sections (**B**) at the peak levels for this contrast are displayed (Right: *x,y,z* = 39, −85, 25; *t = *7.58; Left: *x,y,z* = −39, −85, 16; *t = *5.44). Threshold for these images is set at *p*<0.001 (uncorrected for multiple comparisons). Activation in the angular gyrus is significant at *p*<0.001 (uncorrected for multiple comparisons), cluster size >10 contiguous voxels and also survives SVC at a threshold *p*<0.05 (corrected). Peak coordinates are reported in Montreal Neurological Institute (MNI) space. L = Left side **C,** Condition specific parameter estimates (*β*) in arbitrary units at peak voxel in the right angular gyrus. Grey bars are the conditions used in the scene novelty contrast (Novel_scene>Repeat_scene).

**Figure 5:**
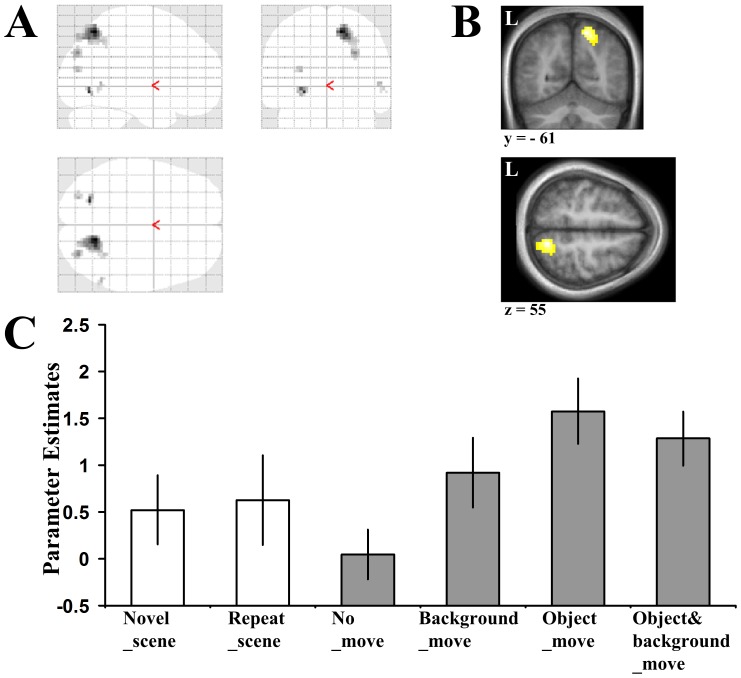
Superior parietal gyrus responds to changes in the spatial relationship between objects and backgrounds. Increased activity was observed in the right superior parietal gyrus when the spatial change conditions and No_scene conditions were contrasted ((Background_move+Object_move+Object&background _move)>No_move). The glass brain (**A**) along with Coronal and Axial sections (**B**) at the peak levels for this contrast are displayed (*x,y,z* = 15, −61, 55; *t = *5.55). Threshold for these images is set at *p*<0.001 (uncorrected for multiple comparisons). Activation in the right superior parietal gyrus is significant at *p*<0.001 (uncorrected for multiple comparisons), cluster size >10 contiguous voxels and also survives SVC at a threshold *p*<0.05 (corrected). Peak coordinates are reported in Montreal Neurological Institute (MNI) space. L = Left side **C,** Condition specific parameter estimates (*β*) in arbitrary units at peak voxel in the right superior parietal gyrus. Grey bars are the conditions used in the spatial change contrast ((Background_move+Object_move+Object&background _move)>No_move).

We examined the response of the AG and SPG to changes in the spatial relationship between objects and backgrounds, and to scene novelty. Our results revealed a dissociation between these brain regions, with the right SPG selectively responsive to changes in the spatial relationship between the object and the background context (i.e. (Background_move+Object_move+Object&background _move)>No_move), and the AG responsive to changes in both the scene (i.e. Novel_scene>Repeat_scene) and the spatial relationship between the object and the background context (see Fig. 4 and Fig. 5). These regions survived a threshold of *p*<0.001 (uncorrected) and a small volume correction at a threshold of *p*<0.05 (family-wise-error corrected for the search volume) (see Methods for details). For completeness, all regions active in our contrasts at a threshold of *p*<0.001 uncorrected are available in Table 1.

**Table 1:** MNI coordinates for peak voxels that showed a significant response in contrasts of interest.

Brain Region	Hemisphere	x	y	z	t value	Cluster size
Novel_scene>Repeat_scene*						
Angular gyrus/intraparietal sulcus	R	39	−85	25	7.58	205
Angular gyrus/intraparietal sulcus	L	−39	−85	16	5.44	115
Parahippocampal cortex	R	33	−37	−17	5.22	110
(Background_move+Object_move +						
Object&background _move) >						
No_move						
Superior parietal gyrus	R	15	−61	55	4.23	157
Posterior transverse collateral sulcus	L	−24	−67	−5	4.99	12
Angular gyrus/intraparietal sulcus	R	30	−79	34	4.30	25
Intraparietal sulcus	L	−27	−79	16	3.99	11
Middle temporal gyrus	R	57	−55	4	3.93	10
Familiar>Unfamiliar						
Angular gyrus/intraparietal sulcus	R	36	−67	28	4.95	40
Parahippocampal cortex	L	−36	−40	−14	4.89	11
Parahippocampal cortex	R	30	−34	−20	4.51	27
Inferior frontal sulcus	R	39	8	28	4.31	19

For each contrast of interest, the MNI coordinates, t values, and cluster sizes for all MTL regions significant at *p*<0.001 (uncorrected for multiple comparisons), cluster size >10 contiguous voxels are listed.

* These coordinates and values are taken from [42] and listed here for comparison.

Significant activation of the right SPG was observed in each of the of spatial change conditions relative to No_move (Background_move>No_move: x = 18, y = −67, z = 46, t-value = 5.58; Object_move>No_move: x = 15, y = −73, z = 55, t-value = 4.29; Object&background_move>No_move: x = 21, y = −64, z = 58, t-value = 4.70). Interestingly, the right SPG was the only brain region to survive a contrast of Background_move>No_move at a threshold of *p*<0.001 (uncorrected). In addition to the main conditions in our spatial change contrast we also examined neural responses to the Scene_move condition. Akin to the Background_move condition when the Scene_move condition was compared with No_move, the right SPG (x = 18, y = −67, z = 46, t-value = 5.58) was the only region to survive our threshold of *p*<0.001 (uncorrected). Critically, while the hippocampus also responds to object-background spatial change [42], its response profile differs from the SPG, in that the hippocampus was more active in the Object_move condition than in the Object&background_move condition. No significant differences in SPG activity were observed for the contrast Object_move>Object&background_move.

#### Dissociation between the angular gyrus and the superior parietal gyrus

Given the differential pattern of responses found in the SPG and AG to object-background spatial change and scene novelty, we next examined the evidence for a more formal dissociation between their response patterns. To consider this issue, we asked whether there was a statistically reliable brain region×contrast (spatial change and scene-novelty) interaction. This was done by extracting each subject’s mean response from the predefined anatomical loci, in relation to the two relevant contrasts: *spatial change novelty* ((Object_move+Background_move+Object&background_move)>No_move) and *scene novelty* (Novel_scene>Repeat_scene) (see Materials and Methods). A 2×2 repeated measures ANOVA yielded a significant brain region×contrast interaction (F_(1,19)_ = 18.04, *p<*0.001) and a significant main effect of contrast (F_(1,19)_ = 5.02, *p = *0.037), but no significant main effect of brain region (F_(1,19)_ <1). Planned pairwise comparisons revealed that the AG was significantly more active in the scene novelty contrast than the SPG (t_(19)_ = 2.38, *p* = 0.028), while the reverse pattern was found in the spatial change novelty contrast (t_(19)_ = −2.44, *p* = 0.025). The effect sizes (measured with Cohen’s d) of the AG’s response to scene novelty and spatial change were 0.74 and 0.27 respectively. The effect size of the SPG’s response to scene novelty and spatial change were −0.05 and 1.04, respectively.

#### Subsequent familiarity for scenes associated with the angular gyrus and parahippocampal cortex

Given that we found that the number of objects recalled by subjects differed across conditions, statistical comparisons following the logic of the scene-novelty and spatial change novelty contrasts were conducted in order to ascertain whether neural responses from the initial functional imaging analysis were related to the cued recall of objects or scene familiarity. These found no differences in object recall (Novel_scene vs. No_move: t_(19)_ = 1.72, *p = *0.101, ((Object_move+Background_move+Object&background_move)>No_move): t_(19)_ = 1.22, *p = *0.239), or scene familiarity scores between conditions (Novel_scene vs. No_move: t_(19)_ = 1.64, *p = *0.118, ((Object_move+Background_move+Object&background_move)>No_move): t_(19)_ = 0.48, *p = *0.638). Thus, we find no evidence that our neural responses from the initial functional imaging analysis were related to the cued recall of objects or subsequent familiarity.

**Figure 6:**
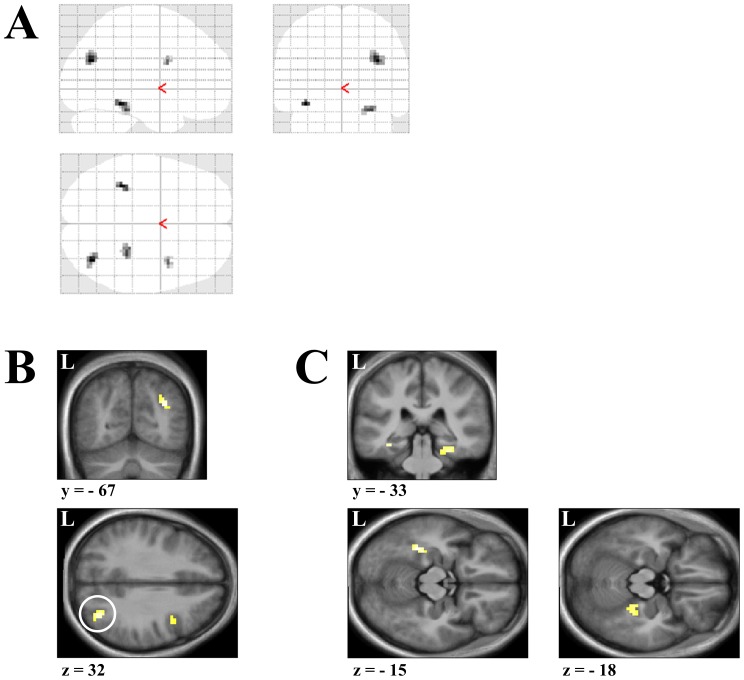
Angular gyrus and parahippocampal cortex respond while viewing pictures containing background scenes that are subsequently classed familiar. Increased activity was observed in the right angular gyrus and bilateral parahippocampal cortex when viewing of subsequently familiar and subsequently unfamiliar scenes was contrasted (Familiar>Unfamiliar). The glass brain (**A**), Coronal and Axial sections for the right angular gyrus (**B**) at the peak levels (*x,y,z* = 36, −67, 28; *t = *4.95), along with Coronal and Axial sections for the bilateral parahippocampal cortex (**C**) at the peak levels (Left: *x,y,z* = −36, −40, −14; *t = *4.89; Right: *x,y,z* = 30, −34, −20; *t = *4.51) for this contrast are displayed. Threshold for these images is set at *p*<0.001 (uncorrected for multiple comparisons). Activations are significant at *p*<0.001 (uncorrected for multiple comparisons), cluster size >10 contiguous voxels and also survives SVC at a threshold *p*<0.05 (corrected). Peak coordinates are reported in Montreal Neurological Institute (MNI) space. L = Left side.

For nineteen participants we were able to generate a voxel-wise, subject-specific general linear model to examine the neural activity associated with viewing scenes during the experimental phase that would subsequently be classed as familiar in the post-scan memory task (see Materials and Methods). Too few objects were recalled in the post-scan task to examine the impact of subsequent cued-recall of the objects across subjects. Comparing the response to pictures where the background scene was subsequently classed ‘Familiar’ with pictures for which the background scene was subsequently classed ‘Unfamiliar’ revealed significantly greater activity in the right AG (extending into the intraparietal sulcus (IPS)) and bilaterally in the parahippocampal cortex (Fig. 6). These regions survived a threshold of *p*<0.001 (uncorrected) and a small volume correction at a threshold of *p*<0.05 (family-wise-error corrected for the search volume) (see Methods for details). For completeness, all regions active in our contrasts at threshold of *p*<0.001 (uncorrected) are available in Table 1. Because some studies have reported indoor scenes to elicit more parahippocampal activity than outdoor scenes [54] we examined whether the subsequent familiarity effect was related to indoor/outdoor scene status. We found no difference in the number of outdoor and indoor scenes judged familiar (t_(18)_ = −1.0, *p = *0.329). Furthermore, we examined whether the subsequent familiarity effect was driven by a particular set of scene stimuli, commonly judged familiar by all subjects. This was not found to be the case; a Shapiro-Wilk test found the distribution of familiarity scores across items did not differ from that expected from a normal distribution (W = 0.996, *p>*0.1). Therefore, our results provide evidence that the parahippocampal cortex and the AG are engaged in successful scene encoding.

#### Increased connectivity between the parahippocampal cortex and angular gyrus during novel scene presentation

**Figure 7:**
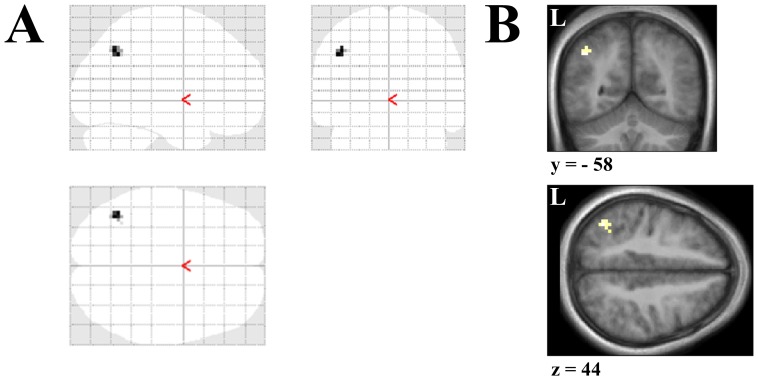
Angular gyrus is functionally connected to the parahippocampal cortex during the viewing of novel scenes. PPI analyses, run on the scene novelty contrast, revealed that the right parahippocampal cortex had an enhanced connectivity with the left angular gyrus. The glass brain (**A**), along with Coronal and Axial sections for the left angular gyrus (**B**) at the peak levels for this contrast are displayed (*x,y,z* = −45, −58, 40; *t = *4.38). Threshold for these images is set at *p*<0.001 (uncorrected for multiple comparisons). Activations are significant at *p*<0.001 (uncorrected for multiple comparisons), cluster size >10 contiguous voxels. Peak coordinates are reported in Montreal Neurological Institute (MNI) space. L = Left side.

Given the reported anatomical relationship between parahippocampal cortex and AG [43] we used two PPI analyses to test for evidence of increased functional connectivity between these regions during the scene novelty and spatial change novelty contrasts (see Materials and Methods). We found that the correlation between activity in the right parahippocampal seed region and the left AG (*x,y,z* = −45, −58, 40; *t = *4.38) was significantly modulated by the scene novelty contrast (Novel_Scene>Repeat_Scene), such that activity in these two regions was correlated when viewing novel scenes but not when viewing repeated scenes (see Fig. 7). Although this AG activity was located outside of the ROI derived from [41], this was the only region significant at a threshold of *p*<0.001 (uncorrected). Furthermore, this AG activity was observed bilaterally at a less conservative threshold, *p*<0.005 (uncorrected). The second PPI analysis showed that the correlation between activity in the right parahippocampal seed region and the left middle occipital gyrus (*x,y,z* = −60, −55, −2; *t = *4.63) was significantly modulated by the spatial change novelty contrast ((Object_move+Background_move+Object&background_move)>No_move), such that activity in these two regions was correlated when viewing scenes containing spatial changes but not when viewing unchanged scenes. However, no significant activations were found in our parietal ROIs, or the rest of the parietal cortex at p<0.001 (uncorrected) in this latter PPI analysis. This was also the case for two further PPI analyses, both of which were conducted using the familiarity contrast (Familiar>Unfamiliar) and seeding in the right and left parahippocampal cortices (regions that were significantly active in the scene familiarity contrast) even at a less conservative threshold of *p*<0.05 (uncorrected). Together, these PPI analyses provide evidence that, within the current data set, the activity in the parahippocampal cortex and the AG were significantly more correlated when subjects viewed novel scenes relative to when they viewed repeated scenes. In contrast, no evidence of changes in correlated activity were observed in relation to spatial change or scene familiarity.

### Experiment 2: Eye-tracking

#### Behavioural results

Subjects performed at 94.8% (SD, 5.7%) accuracy on the target detection task. Their accuracy did not differ significantly from the fMRI participant group in Experiment 1 (t_(34)_ = 0.53, *p* = 0.60).

#### Eye-tracking results

Eye-tracking data were analysed using t-tests, which replicated the same statistical comparisons used in our fMRI design. We previously reported eye-tracking measures for the different conditions in Howard et al. [42]. Analysis of the measures revealed that there were no significant differences between Novel_Scene>Repeat_Scene. Here, in addition to the previous analysis, we examine the data for potential eye-movement differences that correspond with the spatial change contrast ((Object_move+Background_move+Object&background_move)>No_move). Given that the SPG has been linked to saccadic eye movements [55], we specifically examined whether there were differences in saccade amplitude for this spatial change contrast and found no significant differences for this comparison (*p* = 0.68). For completeness, we also examined the number of fixations. For the background ROI analysis the No_move condition elicited significantly more fixations compared with the spatial change conditions (Object_move, Background_move, Object&background_move) (t_14_ = 9.6, *p*<0.001). However, we found no significant differences in the number of fixations across conditions for the current object ROI analysis (*p* = 0.10) or the mean total number of fixations (for entire scenes) (*p* = 0.54) for this comparison.

## Discussion

We used an fMRI repetition paradigm to characterize the contribution of the PPC to processing two main types of change in natural scenes: 1) a change to a novel scene and 2) a change in the object-background spatial relationship within the scene. Our results demonstrate a dissociation between the SPG and the AG: whilst the SPG was selectively engaged by changes to the spatial location of objects and their background context, the AG was responsive to both changes to the scene content and changes in the spatial relationships. Critically, the profiles of responses observed in these two regions were significantly different from one another, evidenced by a dissociation between the magnitude of the response of AG and SPG to scene novelty and spatial change. Further, the diverging response profiles of these two regions of the PPC could not be explained by differences in eye movements obtained in a separate behavioural study. These results provide support for models of parietal function in which dorsal and ventral regions of PPC make separate contributions to processing visual information [21], [56] and help clarify their respective roles in real-world scene processing.

Right SPG was specifically responsive to the changes in the location of the object and background and was not modulated by scene novelty or familiarity. Given the observation that saccadic eye movements are associated with this region [55], and its anatomical links to the superior colliculus [43] one might argue that our observations arise from differences in saccade amplitude. Importantly, however, we found no difference in saccade amplitude between the No_Move and spatial change conditions, suggesting that differences in eye movements are unlikely to account for the observed neural findings. Instead, our findings – in showing that the SPG is sensitive to changes in the position of objects (i.e. Object_move), background (i.e. Background_move), and the entire scene (i.e. Scene_move) – together with previous evidence [24] are consistent with a role for this subregion of PPC in coding visual space in an egocentric-centred framework.

It is worth relating our SPG finding to previous work implicating this subregion of dorsal PPC, and nearby regions such as dorsal IPS (dIPS), in representing information in visual short term memory (VSTM [57]), and playing an important role in the conscious detection of rapidly occurring changes in the environment [58], [59]. For example, a previous study using a change detection paradigm observed that the right SPG exhibited greater activity under conditions when changes were consciously detected than under conditions of change blindness [58]. Further, repetitive transcranial magnetic stimulation at the scalp, above a coordinate similar to the peak activation we report in SPG, impairs change detection, indicating that this region plays a causal role in detecting subtle visual changes [59]. Whilst this set of studies [59], and others (e.g. [60]), have reported that dorsal PPC plays a role in detecting changes in object content, it is interesting to note that it has also been suggested that the dorsal PPC may play a preferential role in coding spatial information (cf object identity in VSTM [61] also see [62]), consistent with the selective response of SPG to spatial changes, and not scene content changes, in our study. Importantly, however, it should be noted that previous work has tended to emphasize the role of the goal-directed nature of the contribution of the dorsal PPC to visual attention [21], VSTM [57] and change detection [58]. In contrast, we demonstrate that the SPG is sensitive to spatial changes under conditions where such information was incidental to the task performed by the subjects (i.e. detection of butterfly target on infrequent trials which were discarded from the analysis). Our findings, therefore, point to the conclusion that the SPG may play a more general role in the spatial coding of the visual environment, even when this information is not relevant to the task at hand.

Viewing visual motion and tracking object motion has been associated with activation in the human MT/V5 and a region encompassing dorsal IPS, SPG [63]–[66]. While our AG region examined here is more dorsal than where MT/V5 is typically mapped to, our SPG region may overlap with the dorsal IPS/SPG region reported in motion processing fMRI studies. Given the short time delay between pictures (250 ms) in our experiment it is possible that our stimuli may be treated as a motion stimulus, not just a re-location on the screen space. Future research specifically manipulating apparent motion during scene view and an incidental task would be useful to understand the contributions of neural populations in dorsal IPS/SPG.

It is also interesting to note that the response profile of the right SPG is similar to that shown by the left hippocampus [42], in that both respond to changes in the spatial relationship between the object and the background, but neither responds to novel scenes. However, these two regions differ in that the left hippocampus was responsive when either the object or background was static while the other changed position, but not when both changed to new locations [42]. Thus, the data support the view that the hippocampus acts to detect associative match-mismatches, generating novelty signals primarily when current input is novel but overlaps sufficiently with past experience to trigger the process of pattern completion [67]–[69]. In contrast, our evidence suggests that the right SPG supports a mechanism that is generally sensitive to spatial changes (e.g. familiarity mechanism), rather than performing specific match-mismatch computations.

Our findings demonstrate that the AG, but not the SPG, reacts to scene novelty. Our observation of increased activity in AG in response to novel rather than repeated scenes is consistent with evidence of activity in the vicinity of this region responding to changes in visual stimulation [41]. Object novelty responses have been observed in this region using fMRI (e.g. [70]) and non-human primate inferior parietal lobe neurons show greater activity to novel images than repeated images [71], [72]. Thus, it is likely that this region is driven by a general change in visual stimuli rather than scene specific stimuli. The response of this region to a change in the spatial relationship between the object and background within the scene provides further support for this view. The absence of any significant differences in the number of saccades or saccade amplitude when novel and repeated scenes were compared indicates that the response of the AG region to scene novelty may relate more to stimulus effects than eye-movement responses.

Previous neuroimaging studies examining scene novelty have focused mainly on the MTL [42], [73]–[80] and have not examined its connectivity with other brain regions. Here, we report that, relative to viewing repeated scenes, viewing novel scenes results not only in increased activity of the parahippocampal cortex and the AG, but also increased functional connectivity between them. Enhanced functional connectivity between these regions, but not between the parahippocampal cortex and SPG, is consistent with diffusion weighted imaging data showing significant anatomical connectivity between parahippocampal cortex and AG, but not the SPG [43]. The current results, therefore, suggest that this anatomical connection serves to route information about scene novelty between parahippocampal cortex and the AG, though interesting we did not find any evidence for a change in neural coupling within these two regions as a function of subsequent memory for scenes.

Our analysis of subsequent familiarity for the scenes allows further characterisation of the relationship between the parahippocampal cortex and the AG. Both regions show increased activity during viewing scenes that were later judged familiar relative to scenes that were judged unfamiliar. Notably, this was not found to be driven by specific scenes, a particular type of content (indoor/outdoor), or by a disproportionate number of scenes from the Novel_scene condition being judged familiar. Whilst subsequent memory effects have been typically associated with regions in the MTL such as the parahippocampal cortex (e.g. [50], [81]), recent evidence has also highlighted their prevalence in the PPC [26], [30]. Interestingly, whilst we observed a positive subsequent memory effect in the AG, such effects are thought to be more prevalent in the dorsal PPC [26]. It is conceivable that our finding may reflect our use of an adaptation paradigm rather than a classical subsequent memory paradigm, or relates to the topographical nature of the stimuli used in this study. One plausible account for our results is that for scenes which subjects attend to the scene layout results in greater subsequent familiarity, with the parahippocampus responsible for processing the scene layout [74], [75] and the AG for modulating attention to the scene [21].

In summary, our data show a dissociation in the response of two posterior parietal regions (AG and SPG) to two different types of change in natural scenes (scene change and spatial change). Our findings provide support for frameworks which emphasize a dorsal-ventral distinction in the function of the PPC, and suggest that the dorsal PPC may support the spatial coding of the visual environment even under conditions where this is task irrelevant. Further, through revealing the differential functional interactions of the SPG and AG with the MTL our results help advance our understanding of how the MTL and PPC cooperate to update representations of the world around us.
